# Mangiferin as New Potential Anti-Cancer Agent and Mangiferin-Integrated Polymer Systems—A Novel Research Direction

**DOI:** 10.3390/biom11010079

**Published:** 2021-01-09

**Authors:** Svetlana N. Morozkina, Thi Hong Nhung Vu, Yuliya E. Generalova, Petr P. Snetkov, Mayya V. Uspenskaya

**Affiliations:** 1 Institute BioEngineering, ITMO University, Kronverkskiy Prospekt, 49A, 197101 Saint-Petersburg, Russia; vuhongnhungs@gmail.com (T.H.N.V.); ppsnetkov@itmo.ru (P.P.S.); mv_uspenskaya@itmo.ru (M.V.U.); 2 Department of Analytical Chemistry, Faculty of Industrial Technology of Dosage Forms, Saint Petersburg State Chemical Pharmaceutical University, Prof. Popova Street 14A, 197022 Saint-Petersburg, Russia; julia.air@mail.ru

**Keywords:** mangiferin, cancer, molecular mechanisms, polymer systems, drug delivery

## Abstract

For a long time, the pharmaceutical industry focused on natural biologically active molecules due to their unique properties, availability and significantly less side-effects. Mangiferin is a naturally occurring C-glucosylxantone that has substantial potential for the treatment of various diseases thanks to its numerous biological activities. Many research studies have proven that mangiferin possesses antioxidant, anti-infection, anti-cancer, anti-diabetic, cardiovascular, neuroprotective properties and it also increases immunity. It is especially important that it has no toxicity. However, mangiferin is not being currently applied to clinical use because its oral bioavailability as well as its absorption in the body are too low. To improve the solubility, enhance the biological action and bioavailability, mangiferin integrated polymer systems have been developed. In this paper, we review molecular mechanisms of anti-cancer action as well as a number of designed polymer-mangiferin systems. Taking together, mangiferin is a very promising anti-cancer molecule with excellent properties and the absence of toxicity.

## 1. Introduction

**Figure 1:**
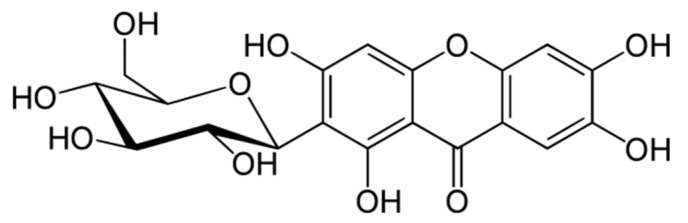
Molecular structure of mangiferin.

Mangiferin, also known as alpizarin or quinomine, belongs to the group of organic class compounds of xanthones. Its chemical formula is C_19_H_18_O_11_ and its average molecular weight is 422.34. Until the 1960s, its structure remained unknown [1,2,3]. Only recently, X-ray diffraction analysis of mangiferin has been conducted [4,5]. It is a polyphenol linked to glucose residues via an abnormal C-C bond (Figure 1).

According to the IUPAC nomenclature, mangiferin is identified as: 1,3,6,7-tetrahydroxy-2-[3,4,5-trihydroxy-6-(hydroxymethyl) oxan-2-yl]-9*H*-xanten-9-one.

In 1908, a pigment—mangiferin—was first isolated from mangoes (*Mangifera indica* L., *Anacardisaceae*) [3,6]. Mangiferin was also found in many other plants, in particular, in the families *Anacardiaceae* and *Gentianaceae* [7]. In mangoes, mangiferin is viable as a crystal in the leaves, cores and rind of the trunk [2,8] and in the pods and seeds of the fruit [8].

Mangiferin is slightly soluble in ethanol, water and insoluble in some non-polar solvents (for example, n-hexane or diethyl ether) [9]. In water, mangiferin solubility is only at 0.111 mg/mL [10]. 

In the traditional medicine of several countries such as China, India and Cuba, mangiferin-rich plants have been grown and actively used to treat many diseases such as cardiovascular diseases, diabetes [11], many types of infection and cancer [7,12,13,14,15].

Numerous studies confirm that mangiferin, through different mechanisms, has various biological activities such as anti-cancer [16,17,18,19,20,21,22,23,24,25,26,27], antioxidant [28,29,30,31,32], anti-inflammatory [33,34], anti-diabetic [35,36,37,38,39,40], cardiovascular protection [41,42,43,44,45,46], neuroprotective [13,40,47,48,49,50], antiviral [51,52,53,54], enhanced immunity [55,56,57,58,59,60], gastroprotective effect [61], analgesic activity [62] and radioprotection, as seen in the experiments on mice [63,64,65]. The neuroprotective effect was patented [66], also anti-allergic properties [67,68] and hepatoprotective activity [69] have been published.

Some preliminary studies indicate that mangiferin has the effect of reducing insulin resistance as well as reducing hyperglycemia in animal models that already have diabetes [70]. Mangiferin reduces triglycerides in animals with high-fat diets [71]. Mangiferin can stimulate sensory enhancement, reverse sedation or depression, which can be caused by lipopolysaccharide or reserpine [72]. The rats were relieved from anxiety, after taking oral mangiferin with the dosage of 100 mg/kg [73]. Pure mangiferin enhances memory [74]. 

The use of mangiferin (100 mg/kg, orally) has significantly reversed scopolamine-induced amnesia in animals with Alzheimer’s disease. It also protects cultured neurons against β-amyloid-induced neurotoxicity. These protective effects of mangiferin may be due to its effect on the inhibition of prostaglandins and leukotriene, which are synthesized by cyclooxygenase and lipoxygenase, respectively [38,75]. 

Drug molecules that are able to treat disease, thanks to the ability to be toxic to damaged cells, are usually toxic to healthy cells but mangiferin has a different nature, it is toxic only to diseased cells without damaging normal cells as well as not having an accumulative effect on the liver or kidneys, on the contrary, it has the protective effect of repairing diseased cells in the liver [76,77,78,79,80] and kidney [37,38]. 

Studies in vitro and in vivo have evaluated that mangiferin has no genotoxic, clastogen, acute or embryotoxic effects. Moreover, mangiferin is capable of resisting genetic toxicity caused by known mutagenic agents [81,82,83,84,85,86,87]. 

Therefore, unlike many kinds of medicine used to treat common non-communicable diseases, mangiferin offers broad-spectrum benefits for a wide range of diseases such as having anti-cancer properties, cardiovascular disease prevention and anti-diabetes properties, it does not even give rise to the development of other diseases [88].

It is also interesting that this C-glycoside can cross traverse the gerbil blood-brain barrier [89] and also cross past the rat blood-retina barrier [90]. Even more important is that the use of oral mangiferin has shown that it is bioavailable in humans, which has been demonstrated by its pharmacokinetics (PK) studies. Research results T_max_ and (t1/2(α)) of mangiferin application in humans are similar to those found in rats. The adult intake of 0.9 g of mangiferin did not report any side effects [91]. LD_50_ of the drug was considered to be 400 mg/kg on mice [63]. The mangiferin concentrations were altered in rat plasma (0.6 to 24 μg/mL) and urine (0.48 to 24 μg/mL) after half-day and one-day reception periods [92]. After taking mangiferin at the dose of 74 mg/kg, its concentration in feces and urine of pigs after 9 and 11 days was 1.4% and 1.6%, respectively [93]. In another study, after intravenous administration at the dose of 50 mg/kg, a decrease in the retinal mangiferin concentration was observed from 6.00 ± 1.50 μg/mL after 0.45 h to 0.30 ± 0.02 μg/mL after 5 h [90].

The first part of our review is the description of molecular mechanisms of the anti-cancer action of mangiferin.

Despite its excellent properties and it being an abundant and inexpensive ingredient, it is regrettable that the current clinical development and use of mangiferin is very limited. The reason for this is that its oral bioavailability is too low, at only 1.2% [94]. The cause of this low bioavailability is thought to be mangiferin possessing only low lipophilic properties, poor permeability through the intestinal membrane and low oral uptake [95].

Improving solubility, permeability and increasing retention time in organs to preserve, improve and maintain the pharmacological effects of mangiferin is very important for its practical applications. In the second part, we overview studies on the development of mangiferin integrated polymer systems.

## 2. Anti-Cancer Effects of Mangiferin and Mechanisms of Anti-Cancer Action

There is a great deal of evidence of mangiferin’s anti-cancer activity against many malignant tumors in in vitro and in vivo models. This action is carried out by mangiferin through a variety of mechanisms. Depending on the type of cancer cell and its growth pathways, mangiferin has different modes of action. Sometimes mangiferin simply disrupts the signaling of transcription factors, sometimes it blocks, stabilizes or activates certain enzymes or specific proteins, or it can also protect DNA from injury. The simultaneous implementation of these activities helps mangiferin easily arrest the cell cycle, promoting apoptosis of many types of cancer. Not only that, but it can also work synergistically with other cancer drugs to increase their activity on chemotherapy-resistant cancers. In the following, the specific mechanisms and pathways of mangiferin’s anti-cancer activities are covered in more detail.

### 2.1. Antioxidant and Anti-Inflammatory Effects of Mangiferin

Oxidative stress and inflammation are the two main factors considered to be the main reasons for cancer development.

It is well known that mangiferin is an effective antioxidant [96,97,98], having anti-inflammatory effects [99]. The first major cause is inflammation caused by reactive oxygen species (ROS) [100]. Mangiferin is a very effective ROS scavenger [101]. It has been shown that the use of mangiferin (1, 10, 100 μg/mL) increases the resistance of red blood cells to hydrogen peroxide-induced ROS [102].

ROS inhibitors have been shown to activate NF-κB (Nuclear Factor k-light-chain-enhancer of activated B cells) in cell lines U-937 (lymphoma), HeLa (cervical cancer), MCF-7 (breast cancer) and IRB3 AN27 (a type of nerve cell of the human fetus) [103,104]. In peritoneal macrophages, mangiferin (10 µg/mL) inhibits phosphorylation of IRAK1 (Interleukin-1 Receptor Activated Kinase 1) resulting in the inhibition of NF-κB expression that is induced by lipopolysaccharides (LPS) and peptidoglycan (PDG) [105]. Furthermore, mangiferin interferes with NF-κB activation through inflammatory genes [19,103].

Mangiferin significantly affects the structure of a large number of genes that are important for the regulation of NF-κB-related inflammation [99]. Mangiferin represses the activities of prostaglandin (PG) endoperoxide synthase 2 (PTGS2, COX-2) and reduces the yield of prostaglandin E2 (PGE2) and possibly prostaglandin D2 (PGD2) [75,106], which can be both pro- and anti-inflammatory [107].

Mangiferin reduces the inflammatory response mainly by intervention in the NF-κB pathway [108]. Mangiferin interferes with different steps in the NF-κB activation pathways, which are classical or alternative. The classical pathway is controlled by the IκB kinase complex and p50, while the alternative pathway is controlled by inhibitors of kappa B kinase (IKKa) and p52. Mangiferin also inhibits other factors including TNFR1—Tumor Necrosis Factor Receptor type-1-Associated Death Domain protein (TRADD), TNFR-Associated Factor 2 (TRAF2), factors of NCK Interacting Kinase (NIK) and IKK that induce Secreted Embryonic Alkaline Phosphatase (SEAP) expression, however, there is no significant effect on p65 which is responsible for the expression of SEAP. In addition, mangiferin also inhibits MAPKs p38, the kinase is modulated with an extracellular signal (ERK) and kinase phosphorylation at the Jun N c terminal [105], thereby reducing the MAPK (Mitogen Activated Protein Kinase) signal [108]. There is already evidence of the anti-tumor effects of mangiferin via multiple signaling pathways, which include nuclear NF-κB signaling and cyclooxygenase-2 (COX-2) protein expression [24]. In anticancer activity, mangiferin induces an apoptosis effect, possibly by activation of caspases. Disorders and imbalances between cell proliferation and apoptosis have been identified as the causes of tumor initiation.

Mangiferin enhances mRNA expression of the Peroxisome Proliferator Activated Receptor Gamma (PPARgamma) gene [109] and minifies the transcriptional activation of COX-2. It has been demonstrated by in vitro studies of the MDA-MB-231 cells that mangiferin may play a beneficial role in modulating the regulation of PPARgamma as well as COX-2 [24].

Mangiferin, in a dose-dependent manner, prevents the depletion of total nucleotides, ATP damage, as well as restored energy charge potential in H_2_O_2_-treated erythrocytes. Mangiferin also protects hepatocytes from free radical-mediated hypoxia/reoxygenation damage by forming a complex of mangiferin: Fe^3+^ complexes and neutralizing free radicals [40,110].

Mangiferin reduces lipid peroxidase and increases levels of antioxidant enzymes so it is also effective in vitro against glycated protein/chelate iron-induced toxicity in human umbilical vein endothelial cells [31].

Numerous data about activity mangiferin in in vitro and in in vivo models have been published.

Mangiferin inhibits the initiation, promotion and metastasis of cancer, by targeting pro-inflammatory transcription factors, growth factors, cell-cycle proteins, cytokines, kinases, adhesion molecules, chemokines and inflammatory enzymes [111], at the same time it also activates the estrogen receptor alpha (ERα) [112].

Its anti-cancer effect is confirmed in mouse models in doses of 5–10 mg/kg (i.p. or i.m.) against ascitic fibrosarcoma in Swiss mice [113], 50 and 100 mg/kg (oral) against Benzo(a)pyrene induced lung carcinogenesis [114,115] and 100 mg/kg (oral) against ER-negative breast cancer [116].

Below we classified available data accordingly to the disease type. Summarized data are presented in Table 1.

### 2.2. Mouse Melanoma

Mangiferin inhibits spontaneous metastasis and tumor growth in the highly metastatic malignant cancer B16BL6 model (mouse melanoma) in doses 50, 100 and 200 mg/kg (mice, orally, 21 days) [117]. Mangiferin also suppresses the nuclear translocation of NF-κB and minimizes the expression of phosphorylated NF-κB-inducing kinase (NIK), inhibitors of kappa B kinase (IKK) and kappa B (IκB) and conversely increases the expression of IκB protein in vivo. In vivo experiments, also confirmed the inhibitory effect of mangiferin on matrix metalloproteinases (MMP) MMP-1, MMP-2, MMP-9 and MMP-14 and very late antigens (VLAs) VLA-4, VLA-5 and VLA-6, which are highly overexpressed in metastatic malignancies. Treatment with mangiferin enhances the expression of cleaved caspase-3, cleaved Poly ADP ribose polymerase-1 (PARP-1), p53 proteins such as p53 upregulated modulator of apoptosis (PUMA—is a key regulator of apoptosis) and phosphorylated p53 proteins, and at the same time, it also reduces the expression of Survivin and Bcl-associated X (Bcl-xL) proteins in vivo. The above results confirm that mangiferin is highly selective in blocking the NF-κB pathway through inhibition of NIK activation, thus inhibiting tumor metastasis and growth. Oral mangiferin administration did not show toxicity since there were no differences in body weight between sham control, tumor control and mangiferin-treated groups, at the same time, it also did not exhibit any side effects at all. 

### 2.3. Acute Myeloid Leukemia

Chemotherapy-induced oxidative damage correlates with the development of secondary malignancies such as acute myeloid leukemia (AML).

Mangiferin possesses antileukemic and preventive effects in HL-60 leukemia cells. Mangiferin activates the G2/M phase cell cycle arrest by modulation of the CDK1 (Cyclin-Dependent Kinase 1)-cyclin B1 signaling pathway in a dose-dependent manner. At higher concentrations, it induces Wee1 mRNA expression, significantly suppressing mRNA expression of Chk1 (Checkpoint kinase 1) and cdc25C, and remarkably inhibits the phosphorylation of Ataxia Telangiectasia and Rad3-related protein (ATR), Chk1, Wee1, Akt and Erk1/2. In addition, mangiferin decreases the activation of cyclin B1 and cdc25C, and protein expression levels of Akt and Wee1 via ATRChk1 [18].

Mangiferin is a dose-dependent and time-dependent agent that increases Nrf2 (Nuclear factor erythroid 2-Related Factor 2) expression and protein stabilization in human HL-60 myeloid leukemia cells in vitro. Furthermore, it also inhibits the proliferation and degradation of blood cells through the increasing of the stability of the Nrf2 protein [118].

In HL-60 cells, mangiferin (50 μM) increases Nrf2 protein accumulation, enhances Nrf2 binding of antioxidant response elements (AREs), modulates NQO1 (NAD(P)H: quinine reductases) expression and restricts intracellular ROS levels. It also lowers oxidative stress and relieves etoposide-induced cytotoxicity in mononuclear human umbilical cord blood cells [119].

An important reason for mangiferin’s ability to reduce DNA damage is that it can activate the Nrf2-ARE pathway [120].

The Nrf2-ARE pathway can protect against the action of chemotherapeutical agents on normal cells. However, overexpression of Nrf2 in cancer cells can promote resistance to therapy. 

Under the action of mangiferin (100 μg/mL), the viability of KG-1 cells, KY821 cells and acute myeloid leukemia cells HL-60 was reduced. There was a simultaneous increase in caspase-3 activity and DNA fragmentation. Mangiferin significantly reduced the nuclear penetration of NF-κB p65, however, there were no changes in the expression of other survival signals, such as extracellular signal-regulated kinase 1/2, protein kinase B and p38 mitogen activated protein kinase. Mangiferin blocks the expressions of Bcl-xL and X-linked inhibitors of apoptosis protein (XIAP) without causing any change in the B Cell Lymphoma-2 (Bcl-2), Bcl-2 associated X protein (Bax) and Bim levels. These results confirmed that mangiferin induces apoptosis by inhibition of the NF-κB activation, expressions of Bcl-xL (B Cell Lymphoma-extra large) and XIAP. In summary, mangiferin may be used as an anti-cancer agent and may also be combined with other anti-cancer drugs to treat acute myeloid leukemia [121]. 

Mangiferin at different concentrations (25–200 µmol/L) in a dose- and time-dependent manner, probably downregulates the expression of the BCR/ABL gene, thereby inhibiting the proliferation of K562 chronic myeloid leukemia cells and inducing apoptosis in the K562 cell line. Inhibition of HL-60 cells occurs by suppressing cell cycle progression at the G2/M phase and enhancing the expression of CDC2 and CCNB1 mRNA [122,123,124].

### 2.4. Glioma Cells

Mangiferin promotes miR-15b and inhibits MMP-7, MMP-9 and EMT (epithelial-to-mesenchymal transition), which significantly limits proliferation and increases apoptosis in U87, U373MG and CRT-MG glioma cells. Mangiferin can also influence VEGF-A (Vascular Endothelial Growth Factor) transcription to modulate angiogenesis via NF-κB [125,126]. 

### 2.5. Prostate Cancer

In the LNCaP prostate carcinoma cells, in androgen-sensitive humans, mangiferin significantly reduces TNFα-induced MMP-9 activity, relieves NF-κB activity and inhibits nuclear translocation of the NF-κB subunits, p65 and p50 [127]. It is known that MMP-7 and MMP-9 are also strong promoters of cancer progression and metastasis of malignant tumor cells [128].

Whereas in PC3 prostate cancer cells, mangiferin promotes apoptosis and induces the caspase-3 activity, significantly reduces Bcl-2 expression levels and enhances miR-182 expression [129].

### 2.6. Colon Carcinoma

Mangiferin causes a reduction of NF-κB activation in HT29 cells rendered resistant to oxaliplatin [19].

In cells of colorectal cancer HT29 and cervical cancer HeLa, mangiferin treatment induces a delay in the S phase, which is an important phase of the cell cycle responsible for DNA synthesis [19].

Mangiferin (0.1% in diet) in rat colon carcinogenesis induced by the carcinogen azoxymethane, significantly inhibits the aberrant crypt foci development in rats, significantly lowers the incidence and multiplicity of intestinal neoplasms and reduces cell proliferation in colonic mucosa [130].

### 2.7. Hepatocellular Carcinoma

Mangiferin has potent cytoprotective and antigenotoxic effects against CdCl_2_ induced toxicity in the HepG2 cell line and may decrease in CdCl_2_ induced reactive oxygen species levels and resultant oxidative stress [131].

Mangiferin inhibits human hepatocellular carcinoma cells BEL-7404 also through G2/M phase cell cycle arrest [132,133].

Mangiferin lowers the levels of total bilirubin, AST, SGPT, SGOT and alkaline phosphatase (ALP) in hepatic damage. DEN (diethylnitrosamine)-treated rats showed decreased levels of total protein, serum albumin and globulin. Mangiferin also lowers the levels of tumor markers carcinoembryonic antigen and alpha-fetoprotein [133].

The antioxidant efficiency of mangiferin in DEN-induced rat liver carcinogenesis was evaluated [134].

Oral administration of mangiferin suppresses orthotopic hepatic tumor growth in vivo. The inhibitory effect of mangiferin was mediated through the transcriptional repression of LEF1 (Lymphoid Enhancer Binding Factor 1) via the β-catenin-independent Wnt pathway, with downregulation of MYC (MYC Proto-Oncogene, BHLH Transcription Factor), axin2, MMP2 and CCND1 [135].

### 2.8. Breast Carcinoma

Mangiferin inhibits the growth of MCF-7 breast cancer cells, in a time-dependent manner by downregulating the CDK1-cyclin Bl signaling pathway and inducing G2/M phase cell-cycle arrest [20]. It induces apoptotic cell death by inhibition of the protein kinase C (PKC)-NF-κB pathway. In vivo experiments performed on the MCF-7 xenograft rat model confirmed these results. The mitochondrial cytochrome C level was reduced under mangiferin treatment, which means that apoptosis can be reduced through the mitochondrial pathway. This pathway also supports the increase of caspase-3, -8, -9 and the decreased expression of procaspase-3, -8, -9 activity. 

In C57BL/6J mice, mangiferin reduces tumor volume by 89.4% with a dose of 100 mg/kg, which is close to the effect of the chemotherapeutic drug cisplatin (91.5%). At this dose, mangiferin extended the lifespan of the treated animals [20].

Mangiferin reduces cell viability, restricts metastatic potential, minifies MMP-7 and -9 expression, reverses EMT and interdicts the β-catenin pathway in breast cancer cell lines. It significantly reduces proliferation, weight and volume of tumors and enhances apoptosis, as well as also reducing the expression levels of MMP-7, MMP-9, β-catenin activity, vimentin and increasing E-cadherin expression in MDA-MB-231 xenograft mice to modulate angiogenesis [116].

In the cell line MDA-MB-231 of triple negative breast cancer, mangiferin suppresses the activation of classical NF-κB by IκB kinases (IKK) α/β via impairing IκB degradation, NF-κB translocation and NF-κB/DNA binding. In addition, mangiferin inhibits additional NF-κB pathways involved in cancer cell survival and resistance to therapy such as c-Jun N-terminal kinases (JNK) 1/2, MEK1, p90 ribosomal s6 kinase and mitogen- and stress-activated protein kinase 1 [22]. Vimang@ and mangiferin, when stimulated by TNF, both have the ability to reduce the production of IL-6 (Interleukin-6) and IL-8 (Interleukin-8), thereby reducing the inflammatory response.

### 2.9. Lung Carcinoma

Mangiferin possesses growth-inhibitory and apoptosis-inducing effects against both A549 cells (25 µg/mL) and in A549 xenograft mice in vivo (100 mg/kg, i.p., two weeks). Mangiferin exhibits anti-cancer properties by inducing G2/M phase cell cycle arrest through the cyclin-dependent kinase 1-cyclin B1 signaling pathway downregulation and apoptotic cell death by inhibiting the PKC-NF-κB pathway [23].

An 18-week diet containing mangiferin (oral, 100 mg/kg) significantly improved the high levels of glycoprotein components, membrane lipid peroxidation and ATPases in animals (male Swiss albino mice) with lung carcinoma. It increased the levels of glutathione (GSH), glutathione transferase (GST), quinone reductase (QR), uridin 5′-diphosphate-glucuronosyl transferase (UDP-GT), catalase (CAT), superoxide dismutase, GSH reductase, GSH peroxidase, vitamin E and vitamin C [114,115].

A study on lymphocytes, polymorphonuclear cells (PMN) and macrophages from B(a)P-treated mice by oral mangiferin twice a week (50 mg/kg and 100 mg/kg) for four weeks confirmed the effects of enhanced lipid peroxidation and decreased activity of catalase and superoxide dismutase. Mangiferin also played an immunoprotective role determined by the reduction of oxidative stress, inducing an intermediate response in lymphocytes, neutrophils and macrophages. The IgG and IgM levels were significantly increased and the IgA level was decreased [136].

The levels of glycoproteins, membrane ATPases and membrane lipid peroxidation were significantly decreased under the action of mangiferin (100 mg/kg) in the benzo(a)pyrene-induced lung carcinogenesis (Male Swiss albino mice) [137].

Adjustment of electron transport chain complexes and of key enzymes in the tricarboxylic acid cycle [30] significantly decreases the levels of polyamines, protein carbonyl, nucleic acid content and lipid peroxidation was found after mangiferin treatment in animals (male Swiss albino mice, 100 mg/kg) [138].

In albino mice with lung carcinoma (caused by benzo(a)pyrene (BaP), 50 mg/kg), the use of mangiferin (100 mg/kg) was shown to decrease the activity of lysosomal enzymes such as β-glucuronidase, acid phosphatase, β-galactosidase and *N*-acetyl glucosaminidase [139].

### 2.10. Other Types of Cancer Diseases

In the human cell line nasopharyngeal carcinoma (CNE2 cells), different doses of mangiferin (from 12.5 to 200 μM) inhibited their proliferation through G2/M phase cell cycle arrest, induced early apoptosis, modulated the mRNA, Bcl-2 protein levels and Bax [140]. 

In human cervical cancer HeLa cells, mangiferin downregulated protein expression of BH3 (interacting domain death agonist), Bcl-2 and pro-caspase-3 and pro-caspase-8, thereby activating caspase-3, -7, -8 and -9, eventually leading to apoptosis [26].

In human neuroblastoma caused by the methylmercury(MeHg)-induced IMR-32 cell line, mangiferin significantly suppressed DNA damage, reduced oxidative stress and inhibited depolarization of the mitochondrial membrane, increased levels of GSH and glutathione S-transferase (GST), resulting in a significant decrease in malondialdehyde formation [141].

### 2.11. Synergic Action 

The synergistic effects of the major chemotherapeutic drugs enhance drug use potential and may allow lower dosages of drugs, thereby reducing toxicity and providing higher selective toxicity to malignant cells, as well as decreasing the extent of side effects [142]. 

Mangiferin enhances the activity of pro-apoptotic agents such as cisplatin, vincristine, doxorubicin, etoposide, Adriamycin and AraC in lymphoma U-937 cells [103].

It was demonstrated that mangiferin enhances the activity of hesperidin, even in low concentration in HeLa cells via increased regulation of the TRADD and TNFR superfamily member 25, which are related to the external apoptotic pathway [143]. 

Mangiferin increases the apoptotic effect of oxaliplatin via NF-kB inhibition in HeLa and HT29 cells [19]. The addition of mangiferin in a 10 µg/mL dose has been investigated in HeLa, HT29 and MCF7 cancer cell lines. This addition of mangiferin reduces oxaliplatin IC_50_ values in HT29 (3.4-fold) and HeLa (1.7-fold) cells. Mangiferin causes a reduction of NF-κB activation in HT29 cells rendered resistant to oxaliplatin.

Mangiferin (25 µg/mL) enhances the antiproliferative effects of cisplatin (12.5 µg/mL) on lung cancer A549 cells. Notably, mangiferin exerts anti-cancer effects in vivo, where it was able to markedly decrease the volume and weight of subcutaneous tumor mass and expand the lifespan (100 mg/kg) of xenograft mice [23].

The higher mangiferin concentrations (10, 25 or 50 μM) together with doxorubicin were able to modulate MCF-7 breast cancer cells via the reduction of their viability and the inhibition of the activity of P-glycoprotein (P-gp) for a period of 96 h [144].

Adverse effects such as the myelosuppression of etoposide were reduced under the action of mangiferin in HL-60 cells, as well as a lack of wild type p53 [116]. 

### 2.12. Summary of Anti-Cancer Activities of Mangiferin

Thus, there is much evidence of the anti-cancer activity of mangiferin against many malignancies in in vitro and in vivo models. The dose usually used in animals is 100 mg/kg. The main molecular pathway responsible for the anti-cancer activity is its interaction with NF-kB on various steps. Mangiferin induces G2/M phase cell cycle arrest through the cyclin-dependent kinase 1-cyclin B1 signaling pathway. Another pathway is the downregulation of MMP-7 and MMP-9, which are responsible for cancer progression and metastasis of malignant tumor cells. Activation of caspases-3, -8, -9 is probably the main pathway leading to apoptosis of cancer cells.

Several studies revealed that the use of mangiferin in combination with other anti-cancer drugs leads to a synergic mode of action and fewer side-effects. These important studies may indicate the way to overcome the resistance to some chemotherapeutic drugs.

## 3. Mangiferin-Integrated Polymer Systems

Although mangiferin was found and formulated in the early to mid-20th century, because of its limited bioavailability, it has not been given much attention. The biological activity of mangiferin is very broad, and its integration into the polymer system is still much less. Studies of this type have only been conducted by a few research groups in less than 10 years in limited numbers using some basic methods, new technologies have not yet been used much. 

In the studies conducted, several techniques to develop mangiferin transport polymer systems have been used including the spray-drying technique, simple solvent evaporation, emulsion solvent evaporation, supercritical antisolvent, thin film sonication, nanoemulsion and Sol-Gel synthesis.

### 3.1. Spray-Drying Technique

In 2013, José Roberto R.de Souza et al. published a study on the spray drying of mangiferin packaging in various polysaccharide polymer materials including citric pectin, pumpkin pectin and chitosan [145]. To test the effect of the nature of the polymer on the quality of the encapsulated product, the authors used the same amount of mangiferin (200 mg) in combination with the same amount of each polysaccharide (2.0 g) with or without Tween 80 surfactant (0.1%). Four formulations were used including SD1 (Citric pectin/mangiferin), SD2 (Citric pectin/mangiferin/Tween 80), SD3 (Pumpkin pectin/mangiferin/Tween 80), SD4 (Chitosan/mangiferin/Tween 80). Encapsulating experiments were carried out in Büchi B-290 with the specifications: air temperatures maintained at 160 °C-inlet and 80 °C-outlet, with a feed rate of 6 mL/min, aspirator volume flow of 35 m^3^/h and air flow of 84 L/h. Productivity for all samples was 65%. 

Products obtained in the form of granules were analyzed by the Fourier transform infrared spectra (FTIR), scanning electron microscopy (SEM), high-performance liquid chromatography electrospray ionization mass spectrometry (HPLC–ESI-MS) and electrospray ionization mass spectrometry (ESI-MS). The results were as follows:

- The largest average diameter was observed in SD3 (15 μm), the smallest was in SD4 (2.9 μm), the difference between the two formulae with the same polymer components SD1 (7.2 μm) and SD2 (10.2 μm) was thought to be due to the presence of Tween 80.
- Tween 80 was also thought to cause the surface of the SD2 particles to be smoother and more uniform than SD1 particles. The SD3 formula gave irregular spherical shape with some set type. Meanwhile, the SD4 were spherical particles with a rough surface.
- Tween 80 used in the SD2 formula also helped to increase the compatibility of mangiferin in the polymer.
- Values of mangiferin concentration in the capsules correlated to the particle sizes: the highest one was in SD3 and the lowest was in SD4. These values were 29, 41, 49 and 16 (μg/mg) respectively for SD1-SD4 formulations.

Explaining these phenomena, the authors suggested that the Tween 80 surfactant has a nonionic nature, which can interact more hydrophobically with the matrix despite the possibility of hydrogen bonding. While chitosan, which is a highly positively charged polysaccharide, has lower retention potential than negatively charged pectins. Thus, the nature of the polysaccharide and surfactant had a significant effect on the capacity of drug retaining during spray-drying.

In 2015, Caroline de G. Sampaio et al. integrated mangiferin into chitosan in the presence of Tween 80 to improve the reduction and removal ability of Cr (VI) [146]. They expected that this spray-drying product will be applied to prevent poison in humans and animals. In this experiment, the authors used 50 mg mangiferin in the solution consisting of 100 mL of 1.0% acetic acid containing 0.5 g of chitosan and 0.1% Tween 80. The air temperatures were maintained at inlet 160 °C and outlet 60 °C for pumping 60% and vacuum flow of 100%. Then, Cr (VI) absorption capacity was also tested. Some conclusions are as follows:

- Similar to the previous research, the majority of particles had a spherical shape, rough surface, heterogeneous size—there were microparticles, nanoparticles, broken particles and some aggregates. The average diameter of microparticles was 2.64 μm (equivalent to the smallest diameter of SD4 in the previous work) and that of nanoparticles was 460.54 nm.
- In nanoparticles, chitosan content was higher than in microparticles. The average mangiferin content was 136 μg/mg of particles (much higher than the SD4 sample in the previous publication).
- When chitosan-mangiferin systems were dissolved in a 1.0% acetic acid solution, the hydraulic average diameter of the particles became 467.7 nm. As such, 1.0% acetic acid promotes the dissolution of chitosan and creates self-assembled nanoparticles.
- In chitosan-mangiferin particles, the chitosan amino group was protonated and Tween 80 had the effect of promoting the formation of intermolecular bonds.
- Comparing the ability to adsorb and remove Cr (VI) with chitosan, the chitosan-mangiferin systems showed the collection at pH values 1–7 (different environments of the gastrointestinal tract) and especially at pH 5.0, the improved efficiency of the system reached 57.34% higher than that of free chitosan.
- Cr (VI) absorption and removal efficiency increased over time and reached a maximum at approximately 120 min and had also shown a rapid effect in removing and reducing Cr (VI) during the first 30 min.

In both studies, successful experiments revealed the factors that affect the characteristics and structure of microscopic particles but only at the synthesis and testing properties, the obtained particles are not homogeneous and not tested on living cells. This opens a lot of research directions for the encapsulation of mangiferin by using the spray-drying technique not only with chitosan, pectin but also with many other bio polymers with or without integrating surfactants for oral, parenteral or topical medications. 

### 3.2. Simple Solvent-Evaporation Technique

To improve mangiferin solubility and permeability, Hequn Ma et al. in 2014 announced the creation of a phospholipid complex, and the oral bioavailability of mangiferin in the complex was 2.3 times higher than that of pure mangiferin [147]. The complex was prepared with mangiferin and Lipoid E80 in a molar ratio of 1:1 with the use and evaporation of ethanol as a solvent. The solubility and adsorption capacity were then evaluated by the authors according to the China Pharmacopoeia 2010 standards. The results show that:

- Mangiferin content of the complex was as high as 35.02% (*w*/*w*).
- In the complex, mangiferin was dispersed evenly, interacting with the polar parts of the phospholipid molecule, the non-polar part of the phospholipid was unchanged, and the hydrogen carbon chains in phospholipids could rotate freely, then encapsulated polar parts of mangiferin.
- In the three media (pH 1.2, pH 6.8 and water), the solubility efficiency of mangiferin from the phospholipid complex was higher than pure mangiferin.
- Compared to pure mangiferin and its physical mixture with phospholipids, mangiferin-phospholipid complexes had higher solubility in water or n-octanol. Especially in n-octanol, the solubility of mangiferin-phospholipid was about 30 times higher, the solubility in water was 1.4 times higher when compared with pure mangiferin and the water-oil partition coefficient of the complex was 6.2 times higher than with mangiferin. As a result, the complex increased mangiferin solubility in the lipid phase, which would increase intestinal permeability thereby increasing the oral bioavailability of mangiferin.
- Compared to pure mangiferin at the duodenum, jejunum, ileum and colon, the mangiferin phospholipid complex had higher rates of constraint of drug absorption at 4.9, 4.8, 5.9 and 18.7 times, respectively, and the effective permeability values were also 11.4, 13.25, 9.2 and 64.6 times higher, respectively.
- Pharmacokinetic studies in mice showed that after administration, comparing the physical mixture of mangiferin and phospholipid, their complex showed that the average C_max_ increased from 180.90 to 377.66 μg/mL, the elimination half-life of mangiferin increased from 4.49 to 9.31 h and the relative bioavailability also increased by 230.00%.

Thus, the introduction of mangiferin into the polymer system led to increased absorption, targeted drug delivery to different targets in the body and improved biological efficiency.

### 3.3. Emulsion Solvent Evaporation Technique

Rungkan Boonnattakorn et al. in 2016 published research about the integration of mangiferin into copolymers of ethylene vinyl acetate and vinyl acetate [148]. In this study, the authors compared the effect of the sorbitan ester active substance (Span^®^20) and Pluronic^®^P 123 on the characteristics of emulsion products created by combining mangiferin with ethylene vinyl acetate solutions containing various vinyl acetate contents (12%, 18%, 25% and 40%). The quality of the product matrices was checked in the following aspects: mangiferin dispersion efficiency, melting point, heat of fusion, oxygen permeability, degree of crystallization, glass transition temperature, tensile strength, elongation at break, grip as well as antioxidant activities, release profile of mangiferin, antioxidant activities, statistical analysis. The results were:

- Surfactants helped mangiferin particles to disperse finely in ethylene vinyl acetate films, preventing them from agglomeration (which is observed in films without surfactants).
- The low molecular weight sorbitan esters consist of the hydrophilic heads and hydrophobic tails, while the high molecular weight polymeric surfactants contain a number of functional groups to interact with the particles. Span^®^20 helped to produce particles that were (39.86 ± 20.71 µm) smaller than the size (82.88 ± 30.05 μm) in the Pluronic^®^P 123 system.
- Compared to Pluronic^®^P 123, Span^®^20 produced smaller particles and more stable microemulsions, the produced films had the same flexibility as ethylene vinyl acetate films without surfactants.
- Vinyl acetate contents had a significant influence on film characteristics: increased degree of crystallization and melting temperature; reduced flexibility and degree of mangiferin release into 95% ethanol with reduced vinyl acetate contents. Vinyl acetate content only mildly affected the glass transition temperature. Oxygen permeability (the highest oxygen barrier), which also played an important role in the suppression of oxidative reactions related to the antioxidant activity, increased with the increasing of vinyl acetate content.
- Compared to the ethylene vinyl acetate films combined with mangiferin without any surfactants, the addition of Span^®^20 only slightly affected the mechanical and barrier characteristics of the films, but markedly increased the mangiferin release from the ethylene vinyl acetate matrix, thereby significantly increasing the activity of antioxidants except in 40% vinyl acetate film.

Thus, the mangiferin release from the ethylene vinyl acetate matrix could be handled by the suitable selection of surfactants and vinyl acetate content.

Most recently, Francisco Fabian Razura-Carmona and colleagues reported on the physical and chemical properties as well as evaluation of the anti-topoisomerase activity of poly (lactic-co-glycolic) nanoparticles (PLGA) containing mangiferin [149]. By design, 15 mg of PLGA (75:25) was integrated with different amounts of mangiferin. There were a total of 15 experiments conducted according to the Box–Behnken design, the best model was selected from linear models, two-factor interaction models and quadratic models due to analysis of variance (ANOVA). Anti-Topoisomerase activity was assessed using *S. cerevisiae* mutations JN362a and JN394, in which JN394 promotes deficiencies in DNA regeneration while JN362a is resistant to DNA repair. The cytotoxicity was assessed on two human cell lines HEPG2 and BEAS-2B (HEPG2 is a type of cells with many features of normal liver cells, extracted from hepatocellular carcinoma. BEAS2B is an immortal non-cancerous epithelial cell line in normal human bronchial epithelium). The authors found that:

- Encapsulation efficiency increased with increasing stirring speed and increasing mangiferin concentration, but the increase in concentration also promoted the saturation of the system leading to reduced efficiency again. According to the results of statistical analysis, the most important factor affecting encapsulation efficiency is speed, followed by the influence of concentration ratio and interaction among them. Accordingly, under optimal encapsulation conditions with 15 mg of PLGA, 300 μg mangiferin was stirred for 10 min with a stirring speed of 6000 rpm and obtained 77% and 97% trap efficiency.
- The optimal formulation was obtained with a response surface methodology. The average size was 176.7 ± 1.021 nm with a polydispersity index value of 0.153, and mangiferin packaging efficiency was about 55%.
- Under in vitro conditions, mangiferin is gradually released over a period of 15 to 180 min under acidic conditions (pH 1.5).
- Fingerprint studies have found a change in the maximum absorption wavelength of both the polymer and the mangiferin. This means that intermolecular bond formation has occurred and also explains the transition of the polymer state from crystal to amorphous.
- This encapsulation product was resistant to in vitro digestion (1.5 h) without the metabolism of healthy cells as well as without modulation of their biological activities.
- The results of the toposiomerase resistance tests have shown that the optimal formulation at 25 μg/mL has anticoagulant activity. Even at a high concentration of 2500 μg/mL, this formulation does not alter morphology and could not reduce the viability of the BEAS-2B and HEPG2 cell lines.

Therefore, the matrix exhibited tolerance to the acidic environment in the stomach, an anti-cancer effect through the anti-topoisomerase I enzyme and at the same time showed its ability to multiply healthy cells. However, this hypothesis has yet to be confirmed by cancer cell studies and is still an interesting research direction.

### 3.4. Supercritical Antisolvent Technique

In 2017, García-Casas and colleagues announced the use of a supercritical antisolvent process with one nozzle (SAS1) and two nozzles (SAS2) to produce micron mangiferin-cellulose acetate phthalate in different material ratios (from 1:1 to 1:10) [150]. In this study, the conditions selected were a 1:3 ratio of dimethyl sulfoxide and acetone solvents, 180 bar pressure and 50 °C temperature. Experiments were conducted according to the following procedures:

- In the SAS1 process, both the polymer and the mangiferin were dissolved in the same solution in different proportions (from 1:1 to 1:10), then it was sprayed into the flask via one nozzle. The CO_2_ flow rate was 30 g/min, the solution flow rate was 5 mL/min and the nozzle diameter 100 μm.
- In the SAS2 process, the polymer and mangiferin solutions were prepared separately and they were sprayed into the same tank through two different nozzles. The SAS2 process was performed under the same conditions.

The solubility and the ability to release mangiferin in a simulation of intestinal fluid and gastric juice were also subsequently examined. The results showed that:

- In the SAS1 process, the average size of particles ranged from 0.25 to 0.41 μm with a narrow particle size distribution, the ratio between the mangiferin and the polymer did not affect the particle size and morphology of the collected precipitate and higher mangiferin loadings were found for the lower proportion of mangiferin in the system.
- In the SAS2 process, two different particle morphologies were formed, consisting of the precipitates formed by a large number of very small mangiferin fibers and microspheres by cellulose acetate phthalate in the range 0.2–1.0 μm. The size of the system increased as its polymer ratio increased.
- After the SAS processes, the systems were in an amorphous state, losing their crystallinity because they could not be restructured into a crystal under the effect of supercritical CO_2_ flow and unmodified mangiferin and polymer chemical change. This helped to improve the solubility of mangiferin in water.
- In two liquids simulating intestinal and gastric juice, after three minutes almost 100% mangiferin-cellulose acetate phthalate was dissolved, greatly improved compared to commercial mangiferin (dissolved about 15% in 3 min and 95% after two hours).
- Investigation of mangiferin release level of SAS1 products showed that the presence of cellulose acetate phthalate slowed the process in gastric juice and did not affect the release in the intestinal juice. However, instead of the polymer ratios in the system, their high flow rate during SAS2 was the factor that reduced the release of mangiferin in the digestive environment.

The authors proposed the use of the SAS process technique for the coating and supplying of mangiferin in the pharmaceutical, cosmetic and nutritional industries.

### 3.5. Nanoemulsion Technique

At the beginning of 2019, María Pleguezuelos-Villa developed emulsions of mangiferin and hyaluronic acid with remarkable therapeutic potential for dermatitis and skin regeneration [151]. In this study, different amounts of hyaluronic acid (of different molecular weights) were encapsulated with or without Transcutol-P. The acute inflammatory test in rats was performed. The results obtained were very positive:

- Nanoemulsions had droplet sizes ranging from 194.5 to 397.9 nm, their mean size was 296 nm with a single dispersion distribution (PI ≤ 0.30). The zeta potential was very negative (<−30 mV). They were all physically stable for up to 30 days under 4 °C storage conditions.
- The mean size and zeta potential of the oil core depend on the molecular weight of hyaluronic acid. Short-chain hyaluronic acid nanoparticles had smaller particle size and more negative zeta potential; they were similar to nanoemulsions without polymers.
- The solubility of mangiferin-phospholipid-polysorbate 80 (0.71 ± 0.03 mg/mL) was higher than that of mangiferin (0.10 ± 0.01 mg/mL) and mangiferin-phospholipid (0.25 ± 0.02 mg/mL). The partition coefficient of mangiferin-phospholipid-polysorbate 80 (5.23 ± 0.17) was also higher than that of mangiferin (0.12 ± 0.01) and mangiferin-phospholipid (2.10 ± 0.12).
- High molecular weight hyaluronic acid formed nanoemulsions with high viscosity. All the nanoemulsions had pseudo-plasticity properties (s~0.40), which were unchanged with or without Transcutol-P.
- The lower molecular weight hyaluronic acid formed the nanoemulsion that had more improvement in mangiferin permeability and it was further improved in the presence of Transcutol-P.
- Compared with the demonstrated hollow nanoemulsion, all mangiferin-containing nanoemulsions strongly inhibited edema and decreased myeloperoxidase activity. Treatment with the mangiferin formula showed a significant improvement in animals’ skin, they exhibited inhibitory edema and leukocytosis activity (*p* < 0.01).

As such, this study suggests that the design and development of nanoemulsions could be a hopeful device for the recovery of inflammatory skin disorders.

### 3.6. Sol-Gel Synthesis Technique

**Figure 2:**
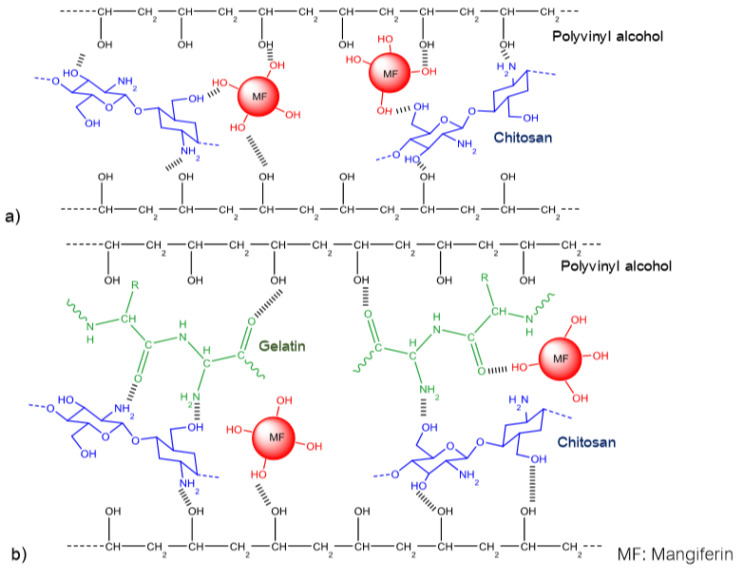
The proposed schematic illustration of inter-molecular and intra-molecular hydrogen bonding in the mangiferin loaded hydrogels: (**a**) binary blend and (**b**) ternary blend [151].

**Figure 3:**
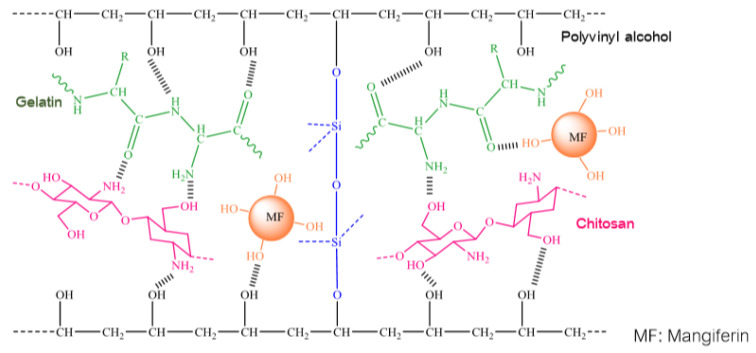
The proposed schematic illustration of intermolecular and intramolecular hydrogen bonding in mangiferin-loaded hybrid-ternary blend hydrogel [151].

Athit Pipattanawarothai in 2019 announced the design of a mangiferin-controlled distribution system using the Sol-Gel synthesis technique [152]. Hydrogel polymers were formed based on the loading of mangiferin into different blending systems of poly vinyl alcohol, chitosan and gelatin including binary (vinyl alcohol, chitosan), ternary (polyvinyl alcohol, chitosan and gelatin) and hybrid-ternary (ternary system associated with siloxane). Polymer systems were tested for mangiferin release at pH values of 5.5 and 10.0 for 8 h. Antimicrobial activity of mangiferin against *Micrococcus luteus 9341* and *Pseudomonas aeruginosa 27853* was tested by diffusion of agar plates. Some interesting results were:

- Similar to other studies on integrating mangiferin into polymer systems, this group of authors has also made a comment about the formation of intra-molecular and inter-molecular hydrogen bonds. However, in this work, by using attenuated total reflection FTIR and SEM with energy dispersive X-ray analysis, the authors not only validated intra-molecular and inter-molecular hydrogen bonds, but also proposed the schemes for interactions between homopolymers and mangiferin as follows (Figure 2 and Figure 3):

Mangiferin is capable of hydrogen bonding with the amide and hydroxyl pendent groups in the homopolymer matrices. Among them, as a polyphenolic, mangiferin prefers to form strong inter-molecular hydrogen bonding between hydroxyl groups and amide and hydroxyl pendent groups of chitosan rather than with PVA’s hydroxyl groups. Gelatin molecules consisting of a multitude of branching and heterocyclic bulk groups increase volume and decrease the strength of hydrogen bonds in hydrogels. Thus, increasing the amount of chitosan may decrease the ability to release mangiferin from the system, especially in a basic medium, and the effect will be the opposite of an increase in gelatin content.

The presence of siloxane bonds complicates the structure and behavior of the hydrogel system due to the nature and ratio of the polymers as well as the degree of siloxane hybridization. In an acidic medium, the hydrogel system swells due to the repulsion of the NH_3_ + cation groups between the molecular chains. However, with the appearance of siloxane bonding, the hybridized network becomes more interlocked with a lower level of swelling. In contrast, in a basic medium, compared to non-hybridized systems, the swelling capacity of the siloxane hybridized network becomes higher due to the high ionic strength of the buffer solution and its hydrophilic properties.

- Thus, the cumulative release of mangiferin from binary and ternary mixtures indicated that the release depends on the hydrogel composition as well as pH. The mangiferin release was lower when its matrix was simpler, either its hydrogel had higher chitosan content or was carried out in a higher pH environment.
- Preliminary results from swelling behavior and in vitro release studies revealed that hydrogel M90PV/5CHI/5GEL-T2 (1.8 g polyvinyl alcohol, 0.1 g chitosan, 0.1 g gelatin, 60 μg siloxane) integrated 0.05 g mangiferin was the most appropriate matrix for the controlled release of mangiferin.

Further studies on cytotoxicity and in vivo release, necessary for the use of this fabricated hydrogel in wound dressings and biomedical applications, have not yet been published.

### 3.7. Thin Film-Sonication Technique

Santi Thanitwatthanasak et al. used the thin film-sonication method to encapsulate two phenols including mangiferin and quercetin in mixtures of Pluronic F127 (F127), Pluronic p123 (P123) and Vitamin E TPGS (TPGS) copolymers [153]. There are 10 runs in the experiment: three single components (F127; P123 and TPGS), three binary mixtures (F127:P123; F127:TPGS and P123:TPGS all with a ratio of 0.5:0.5) and their ternary mixtures (0.76:0.14:0.14 and 0.33:0.33:0.33). 

Encapsulation efficiency, drug loading and micelle concentration were determined and compared with blank micelles using HPLC, DLS, measurement of profile analysis tensiometry. The dissolution tests of the phenolics and phenolic-loaded micelle mixtures were performed under separate simulated gastric and intestinal conditions. All assessments were reviewed for 95% confidence using IBM SPSS Statistics 24 software. A summary of the results shows that:

- Mangiferin loading micelles showed a spherical morphology with a diameter of 14.26 ± 0.52 nm and a zeta potential of −2.89 ± 1.70 mV. The micelle mixtures were neutral particles (±10 mV) with a general feature of high stability against agglomeration. This feature could allow them to diffuse readily through the mucus layer and deeper into the intestinal epithelium. Therefore, mixed micelles were considered stealth bags with special biological stability such as their ability to avoid rapid uptake by the monocytic phagocytic system and maintain circulation time.
- Mangiferin was loaded in both hydrophilic and hydrophobic parts of the micelle mixes by directing its xanthone portion back towards the core and, its glucoside portion was close to corona.

Mangiferin-loaded single copolymers made from F127, P123 and TPGS had an encapsulation efficacy of 91.72%, 75.65% and 92.33%, while ternary blends had a higher encapsulation efficiency of 94–95%. Mangiferin loading efficiency will be higher when combined with micelle systems with hydrophilic copolymer components, F127 and TPGS.

- Analysis of calculations by the Design Expert software obtained the optimal ratio of mangiferin loading into copolymers of F127/P123/TPGS of 0.120/0.328/0.552.
- In the simulated digestive environment, while mangiferin was insoluble, all of its micelles showed excellent solubility and sustainable release.

This study demonstrated that the mixed design has been successfully applied to mangiferin multi-response optimization mixed micelle formulas. The developmental mixed micelles have potential applications for an oral delivery system of drugs with low bioavailability on the basis of nanoparticles.

### 3.8. Summary of the General Characteristics of the Polymer-Mangiferin Systems

Evaluation of differences in the polymer-mangiferin systems is presented in Table 2.

Currently, although the number of studies on mangiferin-integrated polymer systems is very limited, it is obvious that this is a very promising new research direction. The studies only showed some common characteristics of the polymer-mangiferin systems as follows:

- Mangiferin prefers to combine with hydrophilic polymers with smaller molecular sizes than hydrophobic polymers or bulky molecules.
- Mangiferin interacts closely with positively charged polymers (such as chitosan) to facilitate the development of a sustainable polymer system but this made it difficult to release the drug. Research is needed to have an appropriate ratio when combining these polymers with mangiferin.
- Bonding formed in mangiferin loading polymer systems is mainly intermolecular and intra-molecular hydrogen.
- Mangiferin can bind to both hydrophilic and hydrophilic parts of polymer systems, which greatly enhances the encapsulation efficiency and its loading efficiency, and its solubility and permeability are also greatly improved.
- Most of the polymer systems of mangiferin dissolve better in an acidic environment than in a base environment, this solubility could be adjusted by changing the proportion of polymers in the systems.
- Usually, the size of the system increases as the proportion of polymers and molecular sizes of the polymers in its composition increase.
- The surfactant chemical structure also affects the characteristics of mangiferin-polymer systems including particle size, distribution of mangiferin and active ingredients in the matrix or their total properties. The presence of surfactants accelerates the release of active ingredients from the polymer systems.

## 4. Summary

Many studies highlighted numerous biological effects of mangiferin, including its mechanisms of anti-cancer activity against many types of malignancies. However, its low oral bioavailability and low absorption in the body are factors that restrict its clinical use. In this view, the development of new drug delivery systems for mangiferin is an urgent field of research.

Mangiferin is like a precious gift from nature that we did not know of for a long time because its various properties have been studied only in the last few decades. The preservation, improvement and extension of the excellent biological activity of mangiferin in polymer systems need to be further studied both in scale, quantity and quality, such as:

- use in other various encapsulation techniques;
- more complete encapsulation techniques have to be implemented;
- create new polymer systems with higher encapsulation quality, better drug loading and biological effects;
- further in vitro and in vivo experiments, aiming the application of these advances at the treatment of life-threatening diseases in humans.

## Figures and Tables

**Table 1:** Summary of mangiferin targets in different types of cancer.

Type of Cancer	Molecular Targets	Model	Doses, Regime	Reference
Breast cancer	Activation of NF-κB	MCF-7 cell line		[103,104]
	Inhibits P-gp activity	MCF-7	10, 25 or 50 μM	[144]
	Downregulates the CDK1-cyclin Bl signaling pathway; induces G2/M phase cell-cycle arrest; increases caspase-3, -8, -9; decreases expression of procaspase-3, -8, -9 activity	MCF-7 xenograft rat model		[20]
Triple negative breast cancer	Induces decreasing of MMP-7 and -9, and EMT; significantly inhibits the activation of β-catenin pathway; decreases tumor volume, weight and proliferation, increases apoptosis; lowers expression of MMP-7 and -9, vimentin and active β-catenin, and higher expression of E-cadherin	MDA-MB-231 and BT-549 cells; MCF-7 and T47D MDA-MB-231 xenograft mice	300 μM 100 mg/kg	[116]
	Extends lifespan	C57BL/6J mice	100 mg/kg	[20]
Triple negative breast cancer	PPARgamma, COX-2	MDA-MB-231 cell line		[24]
Lymphoma	Activation of NF-κB	U-937 cell line	10 μg/mL	[103,104]
Cervical cancer	Activation of NF-κB; downregulates protein expression of BH3, Bcl 2 and pro-caspase-3 and pro-caspase-8, thereby activating caspase-3, -7, -8 and -9; induces a delay in the S phase	HeLa cell line	10 μg/mL	[26] [19]
Mouse melanoma	Suppresses the nuclear translocation of NF-κB; minimizes the expression of phosphorylated NIK, IKK, IκB; inhibits MMP-1, MMP-2, MMP-9, MMP-14 and VLA-4, VLA-5 and VLA-6; enhances the expression of cleaved caspase-3, cleaved PARP-1, p53 proteins; reduces the expression of Survivin and Bcl-xL proteins in vivo	highly metastatic malignant cancer B16BL6 model	50, 100 and 200 mg/kg (mice, orally, 21 days)	[117]
Acute myeloid leukemia	Activates the G2/M phase cell cycle arrest by modulating CDK1-cyclin B1 signaling pathway; induces Wee1 mRNA expression; significantly suppresses mRNA expression of Chk1 and cdc25C; remarkably inhibits the phosphorylation of Ataxia Telangiectasia and Rad3-related protein (ATR), Chk1, Wee1, Akt and Erk1/2; decreases the activation of cyclin B1 and cdc25C, and protein expression levels of Akt and Wee1; increases Nrf2 expression and protein stabilization; enhances Nrf2 binding of antioxidant response element (ARE); modulates NQO1 expression; restricts intracellular ROS levels; reduces the nuclear penetration of NF-κB p65; blocks the expressions of Bcl-xL and XIAP	HL-60 leukemia cells	50 μM 100 μg/mL	[18] [118,119]
Glioma	Promotes miR-15b; inhibits MMP-7, MMP-9 and EMT	U87, U373MG, CRT-MG cells		[128]
Prostate cancer	Significantly reduces TNFα-induced MMP-9 activity, relieved NF-κB activity, inhibits nuclear translocation of the NF-κB subunits p65 and p50	LNCaP prostate carcinoma cells		[126]
	Promotes apoptosis and induces the caspase-3 activity; significantly reduces Bcl-2 expression levels and enhances miR-182 expression	PC3 human prostate cancer cells	20 µM, 40 µM	[129]
Hepatocellular carcinoma	Enhances the expression of CDC2 and CCNB1 mRNA; β-catenin-independent Wnt pathway, LEF1 gene; downregulation of MYC, axin2, MMP-2 and CCND1; decreases AST, ALT, ALP and LDH levels;	Sprague Dawley rats treated with 0.01% diethylnitrosamine (DEN) MHCC97L, HLF cells; the orthotopic HCC implantation murine model (mice); male albino rats of Wistar strain	80 μM 50 mg/kg/2 days, orally 120 µg/mL 10, 20, 30 mg/kg	[124] [133] [135] [136] [132]
Lung carcinoma	Induces G2/M phase cell cycle arrest through the CDK1-cyclin B1 signaling pathway; inhibits PKC-NF-κB pathway; increases levels of glutathione, catalase (CAT), superoxide dismutase, glutathione reductase, glutathione peroxidase, vitamin E and vitamin C; enhances lipid peroxidation; decreases activity of catalase and superoxide dismutase; decreases the activities of GST, quinone reductase (QR) and uridin 5′-diphosphate-glucuronosyl transferase (UDP-GT); significantly decreases the levels of polyamines, protein carbonyl, nucleic acid content and lipid peroxidation; decreases activity of lysosomal enzymes β-glucuronidase, acidphosphatase, β-galactosidase and N-acetyl glucosaminidase	A549 cells (25 µg/mL), A549 xenograft mice	oral, 100 mg/kg oral, twice a week, 4 weeks	[23,115] [114,115] [136] [138] [139] [136]
Colon cancer	Causes a reduction of NF-kB activation; increases in delay in the S phase; increases Nrf2 and manganese superoxide dismutase (MnSOD)	HT29 HT29, Caco-2, HCT116	2.81 mg/100 g	[19] [130]

**Table 2:** Techniques used and the results obtained.

Number	Technique	Polymer Material	Formulations	Results	Characterization Methods	Reference
1	Spray-drying technique	Citric pectin, pumpkin pectin, chitosan	SD1 (Citric pectin/mangiferin); SD2 (Citric pectin/mangiferin/Tween 80), SD3 (Pumpkin pectin/mangiferin/Tween 80), SD4 (Chitosan/mangiferin/Tween 80)	Size (mangiferin concentration): SD1—7.2 μm (29 μg/mg), SD2—10.2 μm (41 μg/mg), SD3—15 μm (49 μg/mg), SD4—2.9 μm (16 μg/mg)	FTIR SEM HPLC–ESI-MS ESI-MS	[145]
		Chitosan	Chitosan 0.5 g; 100 mL acetic acid 1%; mangiferin 50 mg; Tween 80 0.1%	- Sizes: from nano to micrometers; quantification mangiferin: 136 μg/mg; - Cr(VI) removal pH dependent, maximum at pH 5.0	FTIR, SEM, HPLC-UV, DLS and adsorption studies	[146]
2	Simple solvent-evaporation technique	Lipoid E80 (phospholipid 80%)	Mangiferin/phospholipid (molar ratio 1:1)	Mangiferin-phospholipid complex was in semi-solid state; mangiferin content: 35.02%; solubility increasing: 1.4 times in water; 30 times in n-octanol; oil-water partition coefficient improving: 6.2 times; intestinal permeability was enhanced significantly; - C_max_ increasing: 2.3-fold	IR, SEM, HPLC-UV, DSC	[147]
3	Emulsion solvent evaporation technique	Copolymer of ethylene vinyl acetate and vinyl acetate (12%, 18%, 25%, 40%)	Mangiferin emulsion: toluene + surfactant + mangiferin (in tetrahydrofuran); ethylene vinyl acetate solution: ethylene vinyl acetate containing vinyl acetate + toluene; Mangiferin emulsion + EVA solution ⇨ film	- Increased concentration of vinyl acetate: increased tensile strength and decreased oxygen resistance, increased mangiferin clearance, increased antioxidant activity; - Span^®^20: slightly affects mechanical and barrier properties, significantly increases mangiferin release and antioxidant activity	DSC, MDSC, oxygen permeation analyzer, Instron Testing machine, UV-visible	[148]
		PLGA (75:25), PVA	Aqueous phase: PVA + different quantities of mangiferin. Organic phase: PLGA + dichloromethane. The aqueous phase + the organic phase ⇨ Nanoparticle	Nanoparticle size: 176.7 ± 1.021 nm Polydispersity index: 0.153 Encapsulation efficiency: 55% Entrapment efficiency: 97% Formulation (MG4, 25 µg/mL) had antiproliferative activity; Gastric digestion resistance to 1.5 h; No effect on healthy cells	SEM, UV, X-ray Diffraction, Assessment of Anti-Topoisomerase Activity and Cell Viability	[149]
4	Supercritical antisolvent technique	Cellulose acetate phthalate	Mass ratios of mangiferin/cellulose acetate phthalate from 1:1 to 1:10	Submicron and microparticles: In SAS1, particle size is 0.25–0.41 μm, not affected by the mangiferin ratio; cellulose acetate phthalate retards the release of mangiferin in gastric fluids, has no effect on the release in the intestinal fluids In SAS2, the tiny thread and microsphere size is 0.2–1.0 μm, increases with the mangiferin content; the flow rate of the two injections slows down the mangiferin release	FTIR, nanoSEM, UV, X-ray Diffraction	[150]
5	Nanoemulsion technique	HA of different molecular weights	Aqueous phase: mangiferin + glycerin + HA + water; Oil phase: Lipoid^®^ S75 + polysorbate 8 + tocopherol + almond oil + Transcutol-P; The aqueous phase + the oil phase ⇨ nanoemulsions. NE 0 (control without HA), NE I (HA, high molecular weight), NE II (HA, high molecular weight with TranscutolP), NE III (HA, low molecular weight), NE IV (HA, low molecular weight with Transcutol-P)	- Oil droplets average size 296 nm; monodisperses distribution (PI ≤ 0.30); - zeta potential is −30 mV; - pseudoplastic behavior (s~0.4) in presence of HA; - mangiferin release depends on HA molecular weight; - nanoemulsion permeability is improved with low molecular weight HA in the presence of Transcutol-P; - appropriate anti-inflammatory effect	HPLC, TEM microscope, Photon Correlation Spectroscopy, electrophoretic light scattering in thermostatic cell, FTIR, Rheological measurements, Franz diffuse cells	[151]
6	Sol-Gel synthesis technique	PVA, chitosan and gelatin	PVA, chitosan and gelatin including binary (vinyl alcohol, chitosan), ternary (PVA, chitosan and gelatin) and hybrid-ternary (ternary system associated with siloxane)	Hydrogel M90PV/5CHI/5GEL-T2 (1.8 g PVA, 0.1 g chitosan, 0.1 g gelatin, 60 μg siloxane) integrated 0.05 g mangiferin was the most appropriate matrix to release controlled mangiferin	^1^H-NMR, UV-Vis, SEM-EDX and ATR-FTIR	[152]
7	Thin film-sonication technique	Mixtures of Pluronic F127 (F127), Pluronic p123 (P123), and Vitamin E TPGS (TPGS) copolymers	Mangiferin/copolymer mixed micelles (in the copolymer, total proportion of weight fractions of F127 (X_1_), P123 (X_2_) and TPGS (X_3_) is 1; X_1_ + X_2_ + X_3_ = 1)	Micelles with a spherical morphology; diameter—14.26 ± 0.52 nm; zeta potential—−2.89 ± 1.70 mV; encapsulation efficacy: 91.72 (F127), 75.65 (P123), 92.33% (TPGS); excellent solubility and sustainable release in digestive environment	HPLC, DLS, measurement of profile analysis tensiometry Zetasizer, TEM; FTIR, DSC, NMR	[153]

## Data Availability

please refer to suggested Data Availability Statements in section “MDPI Research Data Policies” at https://www.mdpi.com/ethics.

## Abbreviations

ALP	alkaline phosphatase
AML	acute myeloid leukemia
ARE	antioxidant response element
AST	Aspartate Aminotransferase
ATR	Ataxia Telangiectasia and Rad3-related protein
Bax	Bcl-2 associated X protein
bcl-2	B Cell Lymphoma-2
bcl-xL	B Cell Lymphoma-extra large
Chk1	Checkpoint kinase 1
CDK1	Cyclin-Dependent Kinase 1
COX-2	Cyclooxygenase-2
DEN	diethylnitrosamine
DLS	dynamic light scattering
DSC	differential scanning calorimetry
EMT	Epithelial to Mesenchymal Transition
ERK	Extracellular signal-Regulated Kinase
ESI-MS	electrospray ionization mass spectrometry
FTIR	Fourier transform infrared spectra
GSH	glutathione S-transferase
H_2_O_2_	hydrogen peroxide
HPLC	High-performance liquid chromatography
JNK	c-Jun terminal kinases
IKK-alpha	Inhibitor of NF-kB Kinase subunit-alpha
IKK-beta	Inhibitor of NF-kB Kinase subunit-beta
IL-1R	Interleukin-1 Receptors
IL-6	Interleukin-6
IL-8	Interleukin-8
i.m.	intramuscular route of administration
i.p.	intraperitoneal injection
IRAK1	Interleukin-1 Receptor Activated Kinase 1
LEF1	Lymphoid Enhancer Binding Factor 1
MAPK	Mitogen Activated Protein Kinase
MDSC	modulated differential scanning calorimeter
MMP	matrix metalloproteinase
MYC	MYC Proto-Oncogene, BHLH Transcription Factor
NF-kB	Nuclear Factor k-light-chain-enhancer of activated B cells
NIK	NCK Interacting Kinase
NMR	Nuclear Magnetic Resonance
NQO1	NAD(P)H: quinine reductases
Nrf2	Nuclear factor erythroid 2-Related Factor 2
PARP	Poly ADP ribose polymerase-1
PDG	Peptidoglycan
PG	Prostaglandin
PK	Pharmacokinetics
PKC	protein kinase C
PLGA	poly (lactic-co-glycolic) poly nanoparticles
PMN	polymorphonuclear cells
PPAR	Peroxisome Proliferator Activated Receptor Gamma
PVA	polyvinyl alcohol
QR	quinone reductase
ROS	reactive oxygen species
SAS	supercritical antisolvent process
SEAP	Secreted Embryonic Alkaline Phosphatase
SEM	scanning electron microscope
TEM	transmission electron microscopy
TNF	Tumor Necrosis Factor
TNFR	Tumor Necrosis Factor Receptor
TRADD	TNFR with Tumor Necrosis Factor Receptor type-1-Associated Death Domain protein
UV-Vis	Ultraviolet-visible
VEGF	Vascular Endothelial Growth Factor
VLA	very late antigens
XIAP	X linked Inhibitor of Apoptosis Protein
